# Differentiating the impact of anatomic and non-anatomic liver resection on early recurrence in patients with Hepatocellular Carcinoma

**DOI:** 10.1186/1477-7819-8-43

**Published:** 2010-05-24

**Authors:** Karim M Eltawil, Mark Kidd, Francesco Giovinazzo, Ahmed H Helmy, Ronald R Salem

**Affiliations:** 1 Department of Surgery, Yale University School of Medicine, New Haven, Connecticut, USA; 2 Department of Surgery, Theodor Bilharz Research Institute, Cairo, Egypt

## Abstract

**Background:**

For Hepatocellular Carcinoma (HCC) treated with hepatectomy, the extent of the resection margin remains controversial and data available on its effect on early tumor recurrence are very few and contradictory. The purpose of this study was to compare the impact of the type of resection (anatomic versus non-anatomic) on early intra-hepatic HCC recurrence in patients with solitary HCC and preserved liver function.

**Methods:**

Among 53 patients with similar clinico-pathologic data who underwent curative liver resection for HCC between 2000 and 2006, 28 patients underwent anatomic resection of at least one liver segment and 25 patients underwent limited resection with a margin of at least 1 cm.

**Results:**

After a close follow-up period of 24 months, no difference was detected in recurrence rates between the anatomic (35.7%) and the non-anatomic (40%) groups in either univariate (*p = 0.74*) and multivariate (*p = 0.65*) analysis. Factors contributing to early recurrence were tumor size (*p = 0.012*) and tumor stage including vascular invasion (*p = 0.009*).

**Conclusion:**

The choice of the type of resection for HCC should be based on the maintenance of adequate hepatic reserve. The type of resection (anatomic vs non-anatomic) was found not to be a risk factor for early tumor recurrence.

## Background

HCC is considered the fifth most frequent cancer in the world and the third most common cause of cancer related mortality [1]. Although more common in Asia and Africa, the incidence of HCC is increasing in the Western world [2]. According to the Surveillance and Epidemiology End Results (SEER) registries, the average age adjusted incidence of HCC in the United States increased from 1.3 per 100,000 in 1978-1980 to 6.6 per 100,000 based on cases diagnosed in 2002-2006 from 17 SEER geographic areas [3].

Resection for HCC is a widely accepted safe treatment with a very low operative mortality as a result of advances in surgical techniques and peri-operative management [4]. However, identifying an optimum extent of resection is often difficult due to underlying liver disease such as chronic hepatitis or cirrhosis in most patients [5]. Based on the fact that cirrhotic liver has limited capacity to regenerate [6], many surgeons perform limited resection for HCC, focusing on the preservation of 1 cm or greater tumor-free margin to reduce postoperative liver failure in patients with cirrhosis [7]. Anatomic liver resection is theoretically superior to non-anatomic from the oncologic and anatomic aspects [8], however, this technique is considered technically more demanding and often requires a wider extent of parenchymal sacrifice [4,9]. Additionally, several clinical studies have failed to document any improvement in survival [10-12].

The rate of development of postoperative recurrence after hepatic resection remains high [13]. Early recurrence within 2 years of hepatic resection for HCC is likely to be associated with aggressive tumor biology such as high tumor grade, satellite lesions and microvascular invasion [14].

This retrospective study compares the impact of anatomic and non-anatomic resections on early recurrence in HCC patients over a 2 year period. Other pre and peri-operative factors were also evaluated between the two groups.

## Methods

This study was approved by the Human Investigation Committee (HIC) of Yale University as well as the Ethical Committee of Theodor Bilharz Research Institute (TBRI).

### Patients

Between 2000 and 2006, 53 patients who had a preoperative diagnosis of a single HCC and who underwent hepatectomy at Yale-New Haven Hospital and TBRI-General Hospital were included in the study. The pre-operative investigations included blood chemistry, hepatitis B & C markers, alpha-fetoprotein (AFP), abdominal ultrasonography (US), computed tomography (CT), chest radiography with or without liver biopsy based on the diagnostic criteria of the American Association for the Study of Liver Diseases (AASLD) [15]. All selected patients had compensated cirrhosis with Child-Pugh class A/early B or were non-cirrhotics.

### Patient characteristics

The following clinical variables were compared in the two groups: age, sex, viral markers (Hepatitis B [HB] virus surface antigen, anti-HB core antibody, anti-HB surface antibody, hepatitis C virus antibody), presence or absence of cirrhosis, serum albumin, serum total bilirubin, Child-Pugh classification and serum AFP (Table 1).

**Table 1:** Pre-operative demographic data

Variable	Anatomical	Non-Anatomical	*p*
Age (yr)	62.18 +/- 12.09	57.4 +/- 11.06	ns
Sex (M/F)	21/7	18/7	ns
HBV (y/n)	7/21	5/20	ns
HCV (y/n)	11/17	11/14	ns
HBV+HCV (y/n)	3/25	5/20	ns
Cirrhosis (y/n)	8/20	10/15	ns
Serum albumin (gm/dl)	3.76 +/- 0.34	3.84 +/- 0.37	ns
Serum bilirubin (mg/dl)	0.86 +/- 0.15	0.87 +/- 0.28	ns
Child-Pugh (A/B)	23/5	21/4	ns
AFP (>25/<, = 25)	6/22	9/16	ns

HBV: Hepatitis B virus; HCV: Hepatitis C virus; AFP: Alphafetoprotein; ns: Not significant

### Hepatectomy procedures

The patients were divided into two groups. Anatomic resection (n = 28) was defined as the complete removal of at least 1 Couinaud's segment containing the tumor together with the related portal vein and the corresponding hepatic territory. The appropriate segment margins were identified by intra-operative US after discoloration of the parenchyma after ligation of the corresponding arterial and portal venous branches or both. Non-anatomic resection (n = 25) was defined as the resection of the tumor with a margin of at least 1 cm without regard to segmental, sectional or lobar anatomy. There was no evidence of extra-hepatic metastasis. All patients underwent intraoperative hepatic ultrasonography and were deemed to have resectable tumors at the time of surgery.

### Patient follow-up

The two patients groups were subjected to a close follow-up of 2 years. During this period they underwent clinical, radiologic (abdominal US and triphasic abdominal CT scan) and biologic (serum AFP and liver function tests) evaluations. This assessment was repeated every 3 months throughout the follow-up period.

### End Points

The main end points of the study were: 1- In-hospital mortality, morbidity and length of hospital stay. 2- The detection of early disease recurrence through the 2 year follow-up period. The impact of the type of resection (anatomic vs non-anatomic) on early disease recurrence was studied in the two groups. Other risk factors that could play a role in early tumor recurrence such as the tumor size, TNM staging, vascular invasion, pathologic grading and high AFP values were also assessed.

### Statistical analysis

For continuous variables, data are presented as mean +/- Standard Deviation (SD). Group comparisons were performed using univariate analysis (chi-square test or the student *t *test as appropriate). For multivariate analysis, different factors were correlated with early tumor recurrence, and the SPSS Statistical Software (SPSS 16.0, Chicago, IL) was used for these calculations. Survival (24 months) was calculated using the Kaplan-Meier method (Deltagraph 4.0). A *P *value *< 0.05 *was considered significant.

## Results

There was no difference detected between the 2 groups in terms of clinical and demographic characteristics with respect to age, sex, viral hepatitis markers, and the presence of underlying liver cirrhosis, serum albumin, serum bilirubin, Child-Pugh classification and AFP levels (Table 1).

The tumor pathologic grades were not significantly different between the two groups with only a single poorly differentiated tumor in each group (3.5% of the anatomic group and 4% of the non-anatomic). Two patients in the anatomic group presented with fibrolamellar HCC (FL-HCC) which is a rare variant of HCC that has relatively indolent tumor biology and may carry a better prognosis after complete resection [16].

Using univariate analysis methods, no difference was detected between the two groups in terms of tumor staging. Vascular invasion (T3) occurred in 9 patients *in *the anatomic group (32.1%) and 7 patients in the non-anatomic group (28%). Nodal involvement occurred in 2 patients in the anatomic group (7.1%) and one patient in the non-anatomic group (4%)(Table 2). Diaphragm involvement requiring resection occurred in a single patient in the anatomic group.

**Table 2:** operative variables and peri-operative outcomes

Variable	Anatomical	Non-Anatomical	*P*
**Grade **(*differentiation*) - Well diff.	11 (39.2%)	15 (60%)	ns
	
- Moderately diff.	14 (50%)	9 (36%)	ns
	
- Poorly diff.	1 (3.5%)	1(4%)	ns
	
- Fibrolamellar	2 (7.1%)	0	ns

**TNM Stage: **T1	3 (10.7%)	1 (4%)	ns
	
T2	13(46.4%)	17 (68%)	ns
	
T3(*vascular invasion*)	9 (32.1%)	7 (28%)	ns
	
T4	2 (7.1%)	0	ns
	
N+	2 (7.1%)	1 (4%)	ns

Tumor size	5.9 +/- 2.8	4.1 +/- 1.7	**0.02**

Hospital stay	6.9 +/- 1.5	9.6 +/- 2.7	**0.0004**

Morbidity	6 (21.4%)	7 (28%)	ns

Mortality	1 (3.5%)	2 (8%)	ns

Recurrence	10 (35.7%)	10 (40%)	ns

Yale/TBRI	24/4	4/21	**0.001**

Diff.: differentiated. T1: single tumor < 2 cm without vascular invasion, T2: single tumor > 2 cm without vascular invasion, T3: solitary tumor >2 cm with vascular invasion, T4: tumor involving a major branch or portal or hepatic vein. N: nodal involvement. M: metastasis[17]

The overall morbidity was not statistically significant between anatomic and non-anatomic resections (21.4% vs 28%). This was mainly due to respiratory complications; atalectasis and occasionally respiratory distress. Other morbidities included wound infections, urinary tract infections and intra-abdominal collections which required ultrasound guided drainage. The 30-day mortality rates were not significant between the 2 groups (*p = 0.48*). The three overall mortalities were caused by pulmonary embolism, liver failure and portal hypertension respectively. One occurred in the anatomic group and the other 2 were in the non-anatomic group.

Hospital stay was significantly different between the anatomic and non-anatomic groups (6.9 +/- 1.5 vs 9.6 +/- 2.7) with a *p *value of *0.0004. *This difference is attributed to the surgeon preference as the majority of the non-anatomic resections were performed in TBRI-General Hospital where patients are customarily observed in hospital for longer periods of time. All operative and peri-operative outcomes are shown in (Table 2).

The majority of anatomic resections (85.7%) were performed at Yale-New Haven Hospital while 84% of the non-anatomic resections were performed at TBRI General Hospital (*p = 0.001*) (Table 2). The tumor size & site, the presence of underlying liver cirrhosis and the surgeon's experience are the main factors based on which the type of resection was decided.

### Early tumor recurrence and possible predisposing factors

**Figure 1:**
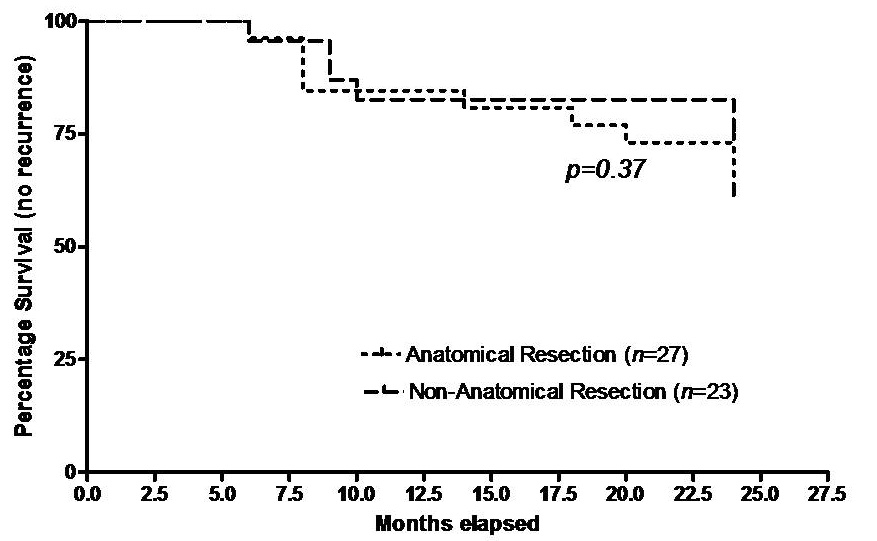
**comparison of intrahepatic recurrence rates between the anatomic and non-anatomic groups through the 24 months follow-up period**.

Using the univariate analysis method, there was no difference between the 2 groups in terms of recurrence through the 24 months follow-up period. The recurrence rate was 35.7% in the anatomic group and 40% in the non-anatomic (*p = 0.74*). This suggests that the type of resection did not have an impact on early recurrence in HCC patients undergoing liver resection (Figure 1).

### Factors affecting early tumor recurrence

Multivariate analysis was undertaken to correlate preoperative demographic data, tumor biologic data and operative variables *with *early tumor recurrence through the 24 months follow-up period in 50 patients after excluding the three early post-operative mortalities (Table 3). To clarify the predictors of early tumor recurrence after hepatectomy, 14 clinicopathologic parameters were analyzed. As a result, variables that affected early recurrence were maximal tumor diameter (Correlation Coefficient [CC] = 0.354; *p *= 0.012) and the tumor T stage which included, in addition to the tumor size, the presence of microscopic vascular invasion as a parameter of the T3 stage [17] (CC = 0.366; *p *= 0.009). The hepatectomy procedure (anatomic vs non-anatomic did not affect early recurrence either in the univariate (*p *= 0.74) or in the multivariate analysis (CC = -0.066; *p *= 0.651).

**Table 3:** Multivariate analysis table correlating risk factors to tumor recurrence using the SPSS Statistical Software.

Recurrence			
**Risk Factors**	**Correlation Coefficient**	***p***	**Number**

Age	0.001	ns	50

Sex	-0.074	ns	50

HBV	0.083	ns	50

HCV	0.016	ns	50

HBV+HCV	0.089	ns	50

Cirrhosis	-0.017	ns	50

Total Bilirubin	-0.248	ns	50

Serum Albumin	0.058	ns	50

Child-Pugh A	0.176	ns	50

Abnormal AFP	0.178	ns	50

Tumor max. diameter	0.354	**0.012**	50

T stage	0.366	**0.009**	50

N stage	0.138	ns	50

Anatomical resection	-0.066	ns	50

## Discussion

HCC has recently gained major clinical interest because of its increasing incidence worldwide and the potential to diagnose and treat the disease at an early stage [18-20]. Although liver transplantation has proven to be an alternative option for the surgical management of HCC in cirrhotic patients, its use is limited by the shortage of donors [21]. Hepatic resection remains the treatment of choice offering the possibility of cure, but the long-term prognosis remains unsatisfactory due to the high recurrence rate [22-24]. Early recurrence is considered one of the most important factors that impact the prognosis of HCC patients [25].

The present study attempts to determine the impact of the type of liver resection (anatomical vs. non-anatomical) on early intrahepatic tumor recurrence in a group of patients with solitary HCC. The patients were similar in preoperative clinical characteristics and tumor biology. The study showed through close follow-up over a 24 months period that the type of resection is not considered a risk factor for early tumor recurrence. On the other hand, other factors such as tumor size and microscopic vascular invasion affected early recurrence. High levels of AFP were not shown to have an impact on early recurrence.

Recent studies have shown that the prognosis of recurrent HCC after resection depends on the time of recurrence, supporting the hypothesis that recurrent tumors are subclinical metastases, originating from the primary tumor and missed during treatment (early recurrence), or de novo HCC arising from persistent fibrosis and hepatitis related carcinogenicity in the remnant liver (late recurrence) [26-28]. In these studies, early recurrence was associated with adverse tumor factors, especially vascular invasion, whereas late recurrence was reported to be primarily associated with the presence of cirrhosis. From these studies, only one study by Imamura *et al. *[27] included the type of resection as a possible risk factor for early recurrence. They concluded that non-anatomic resection is considered a risk factor for early recurrence. However, in this study non-anatomic resection was classified into tumor enucleation and limited resection. The resection margin was not identified in the resection group. In our study all patients undergoing non-anatomic resection had a 1 cm clear margin. A recent study by Cucchetti *et al *[29] compared different risk factors for early and late recurrence in cirrhotic HCC patients. They concluded that the type of resection (anatomical vs limited) is not considered a risk factor for early tumor recurrence which coincides with the results of our study. Although this study considered high AFP levels a risk factor for early recurrence, we attribute this to different cut-off values used for high AFP (60 ng/ml vs 25 ng/ml).

While some authors have found anatomic resection to have a beneficial effect on recurrence-free survival for HCC [30], others have found that anatomic and non-anatomic resection had no significant impact on the risk of tumor recurrence [8,31,32]. These studies were based on overall long-term survival and therefore early and late recurrence risk factors were not taken into consideration.

While some centers pursue a policy of performing non-anatomical hepatic resections whenever possible in order to decrease the rate of postoperative hepatic failure [33], in our study only one patient died from hepatic failure during the early postoperative period. Larger studies may be required to investigate the incidence of post-operative hepatic decompensation following non-anatomic resection.

A recent study by Yoshioka *et al*. [34] predicted early recurrence in HCC after radical resection based on whole human gene expression profiling using microarray analyses. This study concluded that gene expression pattern related to early intrahepatic recurrence inherited in primary HCC can be used for the prediction of prognosis. Although our analysis focused on clinical factors affecting early tumor recurrence mainly the type of resection, further studies based on genetic analysis may provide more evidence regarding the origins of recurrent tumors.

## Conclusion

Hepatic resection for HCC is a balance between the extent of resection and the preservation of hepatic function. The results of this study show that the type of resection (anatomic vs non-anatomic) is not considered a distinct risk factor for early (2 year) tumor recurrence in patients with solitary HCC and preserved liver function. Other factors such as tumor size, staging and pathologic characteristics should be considered predictors of early tumor recurrence.

## Competing interests

The authors declare that they have no competing interests.

## Authors' contributions

All authors have read and approved the manuscript.
